# Adaptive Sport as Complementary and Holistic Health Intervention: Outcomes for Participants to Improve Resiliency, Promote Health, and Live in Recovery

**DOI:** 10.3390/healthcare14020167

**Published:** 2026-01-08

**Authors:** Kaitlin E. Mueller, Derek Whaley, Allie Thomas

**Affiliations:** 1 Psychology, Social Work, Recreational Therapy Department, Slippery Rock University, 1 Morrow Way, Slippery Rock, PA 16057, USA; 2 School of Health and Applied Human Sciences, University of North Carolina Wilmington, 601 S. College Rd., Wilmington, NC 28403, USA; 3 Department of Health and Human Performance, Texas State University, 601 University Dr., San Marcos, TX 78666, USA

**Keywords:** adaptive sports, disability sports, resiliency, health, qualitative, complementary and holistic health

## Abstract

**Highlights:**

**What are the main findings?**

- Adaptive sports should be considered a complementary and holistic intervention to improve resiliency, promote health, and live in recovery.
- Resiliency is built through the disability community fostered within adaptive sports.

**What are the implications of the main findings?**

- Healthcare professionals should promote engagement in adaptive sports for holistic health outcomes, impacting more than just physical health.
- Adaptive sports provide a place for the disability community to flourish while also promoting resiliency for athletes.

**Abstract:**

**Background/Objectives**: Adaptive sports engagement has been strongly studied for physical and social gains for athletes with disabilities, with much less investigation into adaptive sports encompassing holistic health (i.e., reaching domains of physical, social, cognitive, emotional, and spiritual). Therefore, the purpose of this study is to explore adaptive sport participants’ perspectives on their engagement in sport as a complementary and holistic intervention to improve resiliency, promote health, and live in recovery. **Methods**: This study employed a qualitative, phenomenological, and participatory action research design to explore how individuals with disabilities perceive their engagement in adaptive sports. Data were collected from eligible participants across the United States, aged 12 years and older, who provided open-ended responses via survey detailing their adaptive sport experiences. **Results**: Adaptive sport participants (*n* = 47), primarily male (*n* = 26), and White (*n* = 37) with a range of ages 12–75, provided qualitative findings that formed three deductive themes with further inductive subthemes: (1) *Improving Resiliency* highlighting promotive and protective factors supporting resilience development, (2) *Promoting Health* defined by World Health Organization’s holistic health definition, and (3) *Living in Recovery* framed by the Health Protection/Health Promotion Model. **Conclusions**: For this sample of adaptive sport participants across the United States, engagement in adaptive sports is seen as a complementary and holistic health intervention that achieves outcomes beyond just physical and social. Key aspects of adaptive sports were shown to be vital for building resiliency through the disability community environment, improving holistic health, and providing a recovery mindset through new life opportunities.

## 1. Introduction

Living with a disability often involves navigating challenges that affect overall quality of life and wellbeing within one or more domains (i.e., physical, social, emotional, cognitive, or spiritual) [1,2].

To address those challenges, adaptive sport specializes in maximizing athletes’ independent engagement in sports by implementing the least restrictive modifications necessary [3,4]. Adaptive sports serve individuals with various physical disabilities by providing distinct objectives and outcomes through multiple levels of engagement, including recreational, competitive, and elite competitions (i.e., Paralympics) [5] with programs offered in settings such as home and community-based service centers, hospitals, K-12 school systems, and universities [3,6]. Adaptive sports have become essential in providing structured opportunities for physical activity, competition, and social connection tailored to diverse functional needs [7,8,9,10].

Past adaptive sports literature shows efficacy for increasing health outcomes and combating factors of social isolation for individuals with disabilities [3,6,11]. Research also shows adaptive sport engagement assists in mitigating feelings of stigmatization and discrimination while facilitating positive self-image and self-efficacy [3,6,12]. Therefore, adaptive sports continue to gain traction as a trending means to mitigate the effects of disability and promote holistic well-being for athletes to feel empowered to live in recovery [10,13]. To live in recovery can be better defined as an individuals’ ability to be active, engaged, and fully participatory in life, despite congenital or acquired disabilities and subsequent effects of that disability.

For health professionals overseeing adaptive sport programming, best practices include planning programs that are inclusive, evidence-based, and adaptable to the varied needs of the ever-growing disability community [4,14]. Common health professionals that work with athletes in adaptive sports include recreational therapists, athletic trainers, exercise physiologists, physical therapists, occupational therapists, sports medicine doctors, and adapted physical educators [4,15]. Of these allied health professionals, past literature found that recreational therapists are most likely to introduce an athlete to adaptive sports through their services and programs [4,16]. Working interdisciplinary, allied health professionals prescribe and facilitate adaptive sports to help support athletes’ holistic health that includes resiliency, physical health, and perceptions of living in recovery [4].

### Theoretical Background

Theoretical foundations for understanding adaptive sports holistically may have underpinning in the Social Ecological Model of Resilience. According to this model, resilience is defined as an individual’s ability to access and navigate the psychological, social, cultural, and physical resources essential for their holistic health, and their capacity to individually and collectively advocate for these resources to be delivered through paths that are culturally meaningful [17,18]. This model also highlights that adaptive sports help individuals with disabilities build resilience by fostering connectedness and community, enabling them to better adapt to challenges through interactions with social and external systems [19,20].

Additionally, The Health Protection/Health Promotion Model focuses on using active treatment through goal-oriented programming (like adaptive sports) to help clients improve their domains of health (health protection) while pursuing their optimal level of wellness (health promotion) [21]. From the profession of recreational therapy, The Health Protection/Health Promotion Model allows an individual to progress from structured, prescriptive activities for individuals beginning treatment in poor health to self-directed, leisure engagement for those reaching optimal health and recovery [21].

Recent research suggests that allied health professionals like sports medicine doctors and athletic trainers incorporate complementary and alternative medicine into their work with athletes [22,23], but this was not clear when applied to working with adaptive sport athletes. As adaptive sports is often perceived as purely a physical intervention [10], research is lacking when understanding potential holistic health outcomes for athletes who engage in adaptive sports. Further, research has gaps for evidence-based outcomes of adaptive sport from the perspective of adaptive sport athletes (i.e., participatory action research). Therefore, the purpose of this study is to explore how adaptive sport athletes’ perceive their engagement in adaptive sport(s) as a complementary and holistic intervention for promoting resiliency, health, and live-in recovery.

## 2. Methods

This study employed a phenomenological, qualitative, and participatory action research design to explore individuals with disabilities’ perspectives of their engagement in adaptive sport. Participatory action research was used in this study to understand the lived experience of adaptive sport athletes as shown effective in representing the voices of a group illuminate salient aspects of the research process through person-centered inquiry and investigation [24,25]. Data was collected from adaptive sports athletes within the United States. Both the Social Ecological Model of Resilience and The Health Protection/Health Promotion Model underlaid the design of this study by focusing on each athlete’s individual story within the context of investigating complementary and holistic health outcomes.

### 2.1. Participants

This study focused on the perspectives and experiences of adaptive sports athletes’ engagement in adaptive sports. As such, inclusion criteria for this study were as follows: (1) age 12 and above, (2) has participated in an adaptive sports program, clinic, competition, or experience at a minimum of one time as identified on the survey and within the past two years, (3) has self-identified that they have a disability, disease, disorder, or condition, and (4) athlete can answer three of the four cognitive screening questions on the survey. Cognitive screener included orientation questions for month, date, year and participants’ current age.

### 2.2. Data Collection Procedures

Upon IRB approval #8881 at a higher education institution in the United States, the researchers emailed practitioners and community partners who facilitate adaptive sports opportunities to inquire about sending the email and flyer to potential participants who engage in adaptive sports (or their parents/caregivers). Purposive and snowball sampling methods were used, as the email asked the community members and practitioners to send information to other organizations that provide adaptive sport services. Additionally, in-person recruitment involved three members of the research team attending six adaptive sports events to post flyers for athletes in attendance. No incentive was provided to participate in the study, and participation was voluntary. Informed consent was obtained from each participant prior to completing the survey if the participant was over the age of 18. This involved a statement on the front page of the survey identifying the benefits and risks of their participation, with the opportunity to provide their secure signature as consent after reviewing. First, parents/caregivers provided consent with a secure signature. Once completed, the parent/caregiver was provided with an assent form for the eligible minor who reviewed and provided secured signature if desired to participate in the study. After both parties completed the consent and assent forms, the survey was sent to email address or tablet (if completed in person) for the eligible participants.

### 2.3. Instrumentation

Participants completed a 34-item survey of sociodemographic and open-ended questions comprising filling in the blank, choosing all that applied, and open-ended formatted questions. The first survey was developed using expert consultation using three different disciplines of research and practice experts (i.e., athletic training, exercise physiology, and three recreational therapy researchers). After development, the survey was pilot tested, sending it to two recreational therapy practitioners and one adaptive sport athlete. All feedback was reviewed and discussed amongst the researchers, then the final survey was created. Participants used the same survey regardless of disability or age. See Appendix A for the four open-ended survey questions pertinent to the data in this study, as this is part of a larger project for participatory action research with adaptive sport athletes.

### 2.4. Data Analysis

Researchers cleaned the dataset, and five athletes were removed from the data set. One was removed for being under the age of 12; two were removed due to not participating in adaptive sports within the two-year requirement, and two were removed because they were unable to answer the screening questions. Descriptive statistics (frequencies and means) were performed using SPSS version 29 to describe the sample [26].

Deductive thematic analyses were used in Microsoft Excel to analyze the qualitative data collected from open-ended questions and were independently conducted by three researchers [27]. Through the process of deductive analysis, the researchers analyzed data following codes about adaptive sports athletes’ engagement for improving resiliency, promoting health, and living in recovery. Upon completion of individual deductive thematic analyses, researchers met to compare findings, discuss areas of alignment and disagreement, and explore explanations for reasons of difference (i.e., triangulation; [28,29,30] After meeting and discussing individual findings, researchers came to strong agreement on 100 percent of the deductive thematic analysis findings.

Next, data from each deductive theme was individually analyzed for subthemes inductively using thematic analysis following the steps by Braun and Clarke [31]: (1) Data familiarization where the researchers read the data to gain a strong understanding of what participants were describing, (2) Initial code creation using representative quotes and assigning codes using open and axial coding for subthemes by three researchers, (3) Generation of initial themes from data coding from similar meanings that were linked to central concepts, (4) Development and revision of the subthemes where researchers reached consensus on themes and subthemes through cross checking across codes and quotes, (5) Defining and naming each subtheme where subthemes were refined and provided for final name, and (6) Report writing of the themes through selection of my significant quotes to reflect the subthemes.

### 2.5. Trustworthiness

Trustworthiness of the data was established using reflexivity journaling, researcher triangulation, and peer debriefing between all researchers (Jonsen & Jehn, 2009; McMahon & Winch, 2018) [32,33]. Through ongoing and consistent meetings, researchers conducted repeated peer debriefing throughout the entire process to verify that methodological practices were current and properly followed, with in-depth discussions for inter-coder agreement.

## 3. Results

A total of 47 individuals who engage in adaptive sports met the inclusion criteria and participated in this study from across the United States. See Table 1 for participant characteristics of their adaptive sports experiences. Participants were primarily male (*n* = 26) and White (*n* = 37) with a range of ages from 12 to 75. For the participants in this study, the most prevalent adaptive sports were wheelchair basketball, followed by wheelchair tennis, and then adaptive snow skiing.

Deductive thematic analysis produced results organized into three themes that align with adaptive sports as a complementary and holistic health intervention: (1) *Improving Resiliency*, (2) *Promoting Health*, and (3) *Living in Recovery*. Deductive thematic analysis resulted in a subtheme or subthemes for each theme.

(1) *Improving Resiliency*

Participants’ responses as participatory action research data were analyzed through the lens of the Social Ecological Model of Resilience. When inquiring about how adaptive sports improved individual resiliency, findings are focused on attributes that identify strengths in promotive and protective factors, as well as healthy development of resiliency. Two subthemes emerged from heavily saturated data about resiliency: (1) *The Disability Community* and (2) *Positive Social Environments*.

*The Disability Community.*

Adaptive sports provide participants with connections to individuals with similar characteristics to themselves. These social interactions created strong positive impressions and laid the foundation for the development of promotive factors of resiliency in this study. Regarding the connections and united social support made from being part of the disability community, Noah, a young adult who has a spinal cord injury, stated

*Socially, adaptive sports has opened my eyes to a new world of support and encouragement where… I’ve met many friends and feel welcomed into the community of people of all ages and abilities. I have people to turn to when I have questions or need help. Also, I met my partner through adaptive sports, so I’m very grateful for deciding to get involved all those years ago!*

Mateo, who is an older adult with a spinal cord injury, highlighted the importance of shared experiences, describing “I meet other supportive people where we can share our pains and our successes”.

Additionally, participants described how adaptive sports fostered protective factors of resiliency that improved their psychological wellbeing and self-efficacy. Noah directly shared this sentiment about adaptive shooting and track and field when stating:

*The relationships I have formed with other like-minded people who are incredibly resilient have really helped me. I have more to be excited for, and people in my life who support me doing adaptive sports (which is important to me as someone with little family support).*

Many participants echoed Noah in that the positive social component of adaptive sports was crucial to helping them recognize their own capabilities. As another protective factor of resiliency, Mia, who has a lower extremity amputation, shared how the adaptive sports community positively contributes to their mental health and motivation when engaging in weightlifting and CrossFit:

*When surrounded [by adaptive sport athletes], those people continually push themselves to be better… once you [engage with adaptive sport athletes], there is no turning back. When I choose to be around people who want to be better every day, it’s the best kind of infectious and we are capable of so much more together.*

*Positive Social Environments.*

The data also strongly displayed that adaptive sports gives athletes a positive social environment where they could focus on play rather than their disability. These adaptive sport opportunities provided a sense inclusion, allowing athletes to participate freely alongside peers, as Halo, an adolescent who is legally blind, explained, “being able to play and play with others and feeling like a normal kid”. Mateo also shared, “the ability to compete in sports with others without being told that I can’t do that because of my disability”, and Halo echoed similar as adaptive sports “helps me feel normal and part of a team”.

In addition to fostering a sense of inclusion, participants emphasized how adaptive sports foster positive social environments that bring people together to promote a sense of belonging. Scottie shared that “adaptive sports provide commonality between athletes and a since of camaraderie with all involved”, and Emma, an adolescent with cerebral palsy said that “[*adaptive* sports] has made a difference in my confidence and feeling of belonging”. Another participant Bryer, identified the importance of belonging stating that “living in [a remote community] isn’t so easy, [but] sports has helped me feel like I belong and have people with similar issues in my life makes me feel not so alone.” These statements underscore how adaptive sports create environments that build community, foster mutual understanding, and cultivate a shared identity for individuals with disabilities.

(2) *Promoting Health*

The data for the second category of *Promoting Health* was framed through World Health Organization’s definition of health as “a state of complete physical, mental, and social well-being and not merely the absence of disease or infirmity” (World Health Organization, n.d.) [34]. Two subthemes emerged from this theme: (1) *Holistic Physical Health* and (2) *Physical Health Equals Mental Health.*

*Holistic Physical Health.*

Participants consistently reported that adaptive sports improved their overall physical health and independence, especially noting gains in cardiovascular health, strength, endurance, flexibility, and weight loss. Mateo shared that adaptive sports “gets me out of bed and allows me to improve my cardio”, while Theodore, an active older adult with tetraplegia, reported that “[adaptive sports] has greatly improved my physical health… my fitness, strength, endurance, and movement have improved exponentially”. Sophia, an adult with tumors on the spine, identified that adaptive sports provide a structured exercise experience which “is the best thing for me since I am in a wheelchair.”

Participants also identified how training in adaptive sports improved their muscle strength, which translated to greater independence in their day-to-day life experiences. Noah said that “I’ve gained a lot of muscle from pushing in wheelchair racing which makes getting around in my day chair easier”. Mia elaborated on this when sharing “I was morbidly obese and by actively participating in fitness I am at a healthy weight. The stronger my body becomes the easier it is to live an independent life”. Participants frequently reported that the physical benefits gained from adaptive sports improved their lives holistically, reaching far beyond the context of the sport.

*Physical Health Equals Mental Health.*

Corresponding with holistic physical health benefits, participants heavily emphasized the resulting gains in their overall physical and mental well-being through adaptive sports. Many highlighted the importance of consistent engagement in adaptive sports as both a physical benefit but also a tool for improving mood and managing symptoms of depression and stress. Azaiah, an adult with a spinal cord injury who reported engaging in six different adaptive sports, exemplified this when saying, “I like the way [adaptive sports engagement] makes me feel, both mentally and physically after”. Scottie, an adult with an upper extremity amputation, shared that “[adaptive sports] has helped me become a lifelong athlete. My overall health has been better off for it”.

Participants also specifically identified adaptive sports to increase mood regulation, as described by Marjorie that adaptive sport engagement “increases endorphins, [my] peer interaction and fun… good for my mental and physical health”. Other participants highlighted the positive impacts of adaptive sports experiences regarding stress reduction, particularly in outdoor and natural settings, like Colsen who said that adaptive sports are “very important to be active to reduce stress and get me outside to enjoy nature”. Theodore shared similarly that “being active [in adaptive sports] certainly helps your mental state. It keeps you positive and engaged in life”.

(3) *Living in Recovery*

*Living in Recovery* provided a strong theme through the lens of recovery from the Health Protection/Health Promotion Model (Austin, 1998) [21]. When inquiring about how adaptive sports improved individual recovery, findings were saturated from attributes that enhanced optimal health and recovery. One subtheme emerged for this theme: *Opportunity.*

*Opportunity.*

Participants emphasized the importance of adaptive sports and the meaningful opportunities to be active, engaged, and fully participate in life. Further, adaptive sports gave participants the ability to engage or re-engage in sports previously inaccessible to them, effectively opening new doors. For example, Colsen said that adaptive sports provided the “opportunity to be active and involved in outdoor activities”. Bryer identified the importance of “getting back out there.” Emma, as a school-aged participant with cerebral palsy, further shared the opportunity warranted through adaptive sports when saying “I enjoy being able to participate in the same sports that others in my school are participating in”. Participants frequently described feelings of freedom experienced when engaging in adaptive sports like Mateo when sharing, “the ability to compete in sports with others without being told that I can’t do that because of my disability”. Elijah, a young adult with a spinal cord injury, shared that while engaged in adaptive sports they feel “freedom from the disability for a while.”

Furthermore, participants reported that adaptive sports were not only a physical health outlet, but the opportunity to experience life fully. Bryer, an adult with spina bifida, proclaimed that because of adaptive sports “I am no longer wasting away, depressed about not being able to be active”. Adaptive sports provided athletes with opportunity beyond sports as Mateo shared, “I have friends all over the world now that I talk to frequently to share experiences and to plan on meeting at events”, while Theodore reported that adaptive sports has provided the opportunity “for me to live a healthy lifestyle as it has dramatically improved my function.” Overall, adaptive sports provided participants with the opportunity to live in recovery from a holistic lens, impacting both their athletic endeavors and everyday life.

## 4. Discussion

This participatory action research study explored individuals with disabilities’ perspectives of their engagement in adaptive sport, a holistic intervention to promote resiliency, improve health, and live in recovery. To the author’s knowledge, this is the first study suggesting adaptive sports as complementary and holistic health intervention. This study identified several areas of discussion regarding adaptive sports fostering resiliency, serving as an effective and powerful tool for promoting health, and facilitating opportunities to live in recovery.

Viewed through the Social Ecological Model of Resilience, these findings demonstrate how both promotive and protective factors of resiliency (i.e., engagement in adaptive sports) are activated and reinforced through engagement in adaptive sport settings. Participants in this study strongly through the first theme of improving resiliency stated that the disability community of adaptive sports profoundly impacted their ability to be resilient through both sport activities and in everyday life. The disability community formed positive social connections highlighting promotive factors of resiliency that adaptive sport athletes experience when pushing to seek more out of life, both individually and as a social community. Like other research, participants previously shared that their quality of life increases with engagement in adaptive sports [12,35], but these present findings highlight that this drive for life continues into everyday activities because of positive engagement in adaptive sports. Two prior studies addressed resiliency with adaptive sport athletes, but one only included Para badminton athletes [13] and the other included both participants with physical and intellectual disabilities engaging in adapted sport [36]. Therefore, the findings from this study highlight adaptive sports as a complementary and holistic intervention to intentionally build resiliency for athletes. This increase in resiliency stems from the power of inclusion in the disability community as a positive social environment.

Findings from the second theme also indicated that adaptive sports participation serves as a powerful mechanism for promoting health. This finding aligns closely with the World Health Organization’s holistic definition of health as encompassing physical, mental, and social dimensions rather than the mere absence of illness [34]. Participants in this study consistently shared adaptive sports as vital for improving their physical health in a holistic manner by incorporating social and mental aspects into their sporting experiences. Prior research primarily focuses on adaptive sports for purely physical gains [37], yet these present findings highlight the ability for adaptive sports to also impact holistic health. Participants emphasized the role of adaptive sports for improving physical health (i.e., strength, endurance, flexibility, cardiovascular health, and weight loss) alongside the development of the mind–body connection as a result of their sport engagement. This concept of the mind–body connection through physical interventions is well researched, but appears to be a newer finding for applying the mind–body connection to engagement in adaptive sport [38,39,40,41]. Understanding this connection between mental and physical health as a result of this study further demonstrates adaptive sports as a complementary and holistic intervention [41].

Aligning with the concept of the mind–body connection to increase health, engagement in adaptive sports was additionally found to help athletes live in recovery. Results from this third theme showed that the live-in recovery concept revolved around the opportunity for adaptive sport athletes to re-engage in life, ultimately experiencing a greater sense of well-being and recovery. These results stem from the theoretical underpinnings of the Health Protection/Health Promotion Model [21] that show engagement in health promoting interventions can lead an individual from needing dependence to meaningful engagement in life with optimal health.

### 4.1. Implications for Healthcare Professionals

Recommendations for implications for healthcare professionals include foremost considering adaptive sports as a complementary and holistic intervention, with the potential outcomes of increasing resiliency, promoting health, and providing opportunities to live in recovery. Healthcare professionals who work with individuals with disabilities are aware that resiliency has been shown to be crucial for recovery and overall wellbeing [42,43]. So, healthcare professionals, such as recreational therapists, exercise physiologists, athletic trainers, occupational therapists, physical therapists, and sports medicine doctors, should be implementing adaptive sports as more than physical intervention, but as complementary outcomes to promote holistic health and well-being [4]. Secondly, these findings show that adaptive sports allow individuals with disabilities to live in recovery in their communities while engaging in a preferred leisure pursuit. Adaptive sports operate in broader ecological systems, more than purely at the interpersonal level of the individual. For example, families, schools, rehabilitation hospitals, and long-term care facilities can use adaptive sports to promote holistic health and resilience. Healthcare professionals should continue to use adaptive sports to help individuals with disabilities feel included in their communities as a means to live in recovery.

### 4.2. Limitations and Future Research

Despite these strong findings of adaptive sports as complementary and holistic interventions, limitations persist within this study. For example, snowball sampling may have unintentionally excluded the perspectives of adaptive sport athletes, and future research should consider larger sample sizes. Additionally, this study includes data from adaptive sport athletes of multiple diagnoses who engage in varying sports. Furthermore, the study lacked methodical means of triangulation, such as semi-structured interviews, focus groups, or observational data. Additionally, the cross-sectional design of the study limits conclusions regarding recovery trajectories. Future research should intentionally target certain sports (wheelchair basketball, track and field, wheelchair tennis, etc.) for specific diagnoses, using quantitative, qualitative, and mixed methodologies. Coincidingly, future exploration should include deeper analysis of physical benefits for more specificity (i.e., muscular strength, flexibility, endurance, balance) which were initial findings in the present study, yes not saturated fully to form themes. As a final limitation, the data in this study relies on the personal stories and perceptions of adaptive sport athletes who have differing life experiences, personality types, social support, and socioeconomic backgrounds.

## 5. Conclusions

This study continues to add to the body of literature by conceptualizing adaptive sport(s) as a complementary, holistic health intervention rooted in athletes’ lived experiences. Guided by the Social Ecological Model of Resilience and the Health Protection/Health Promotion Model, findings provide evidence that adaptive sports simultaneously facilitate resilience, support holistic health, and promote athletes’ living in recovery. Regarding this study, a holistic health intervention is defined as an intentional, person-centered adaptive sport experience that addresses health domains of physical, mental, social, emotional, and spiritual domains while facilitating promotive and protective factors of resilience. Furthermore, results from this study support the integration of adaptive sports by healthcare professionals as a viable, evidenced-based intervention for promoting resilience, holistic health, and recovery among individuals with disabilities.

## Figures and Tables

**Table 1:** Characteristics of Adaptive Sport Participants.

Age	*n*	Current Adaptive Sports Participation	Disability
12–18	5	Soccer, tennis, track and field, wheelchair basketball	Cerebral palsy, congenital fiber type disproportion, legally blind, spinal cord injury (unspecified), traumatic brain injury
19–25	4	Adaptive shooting, adaptive snow skiing, adaptive water skiing, power soccer, tennis, track and field	Cerebral palsy, spina bifida, spinal cord injury (not specified)
26–30	5	Adaptive kayaking, basketball, cheer, CrossFit, goalball, golf, pickleball, sled hockey, soccer, track and field, weightlifting, wheelchair basketball, wheelchair rugby, wheelchair softball, wheelchair tennis	Cerebral palsy, Guillan Barre Syndrome, right above knee amputation
31–35	3	Adaptive kayaking, Jiujitsu, wheelchair basketball	Spinal cord injury (not specified), Spinal cord injury (T6 Asia A complete)
36–40	6	Adaptive kayaking, adaptive snow skiing, adaptive shooting, pickleball, sled hockey, tennis, track and field, wheelchair basketball, wheelchair football, wheelchair rugby, wheelchair soccer, wheelchair softball, wheelchair tennis	Cerebral palsy, Rett syndrome, spina bifida, spinal cord injury (not specified)
41–45	4	Adaptive snow skiing, cycling, softball, wheelchair basketball, wheelchair tennis	Cerebral palsy, seizure disorder, spina bifida
46–50	4	Adaptive kayaking, adaptive snow skiing, handcycling, horseback riding, fishing, hunting, mountain biking, pickleball, sled hockey, soccer, surfing, utility terrain vehicle riding, track and field, wheelchair basketball, wheelchair rugby, wheelchair tennis, whitewater rafting	Spinal cord injury (not specified), spinal cord injury (T9)
51–55	4	Adaptive shooting, archery, curling, pickleball, track and field, wheelchair basketball, wheelchair football, wheelchair rugby, wheelchair soccer, wheelchair softball, wheelchair tennis	Below knee amputation, paraplegic (not specified), spinal cord injury (not specified)
56–60	2	Adaptive snow skiing, cycling	Below elbow amputation, spinal cord injury (not specified)
61+	10	Adaptive cycling, adaptive kayaking, adaptive shooting, adaptive snow skiing, beep baseball, boccia, goalball, golf, pickleball, sit skiing, sit volleyball, sled hockey, softball, tennis, track and field, triathlon, wheelchair basketball, wheelchair rugby, wheelchair softball, wheelchair tennis	Cerebral palsy, end stage osteoarthritis, hip replacement, left below knee amputation, right above knee amputation, spinal cord injury (not specified), tetraplegia C7, tumors on spine T12-L3

Note: *n* = 47. In column titled *Current Adaptive Sports Participation*, the sport was only listed once as opposed to each time a participant stated they engaged in that sport. In column titled *Disability*, the disability was only reported once, as opposed to each time a participant reported the same disability as a previous participant.

## Data Availability

The datasets presented in this article are not readily available as this is part of an ongoing study with additional publications in progress. Requests to access the datasets should be directed to kc37@txstate.edu.

## Appendix A

Qualitative Questions pertinent to this study

1. In as much detail as you want, explain what you enjoy most about doing adaptive sports? If nothing, please explain what you do not enjoy.
2. In as much detail as you want; explain how adaptive sport(s) has improved your physical health (for example, fitness, strength, endurance, breathing, movement)? If nothing, please explain how it has not improved your physical health.
3. In as much detail as you want; Explain how adaptive sport(s) has improved your mental health (for example, anxiety, depression, sadness, isolation, happiness, excitement)? If nothing, please explain how it has not improved your psychological wellbeing (positive feelings like happiness and satisfaction).
4. In as much detail as you want; Explain how adaptive sport(s) have improved your social opportunities (for example, social life, friendships, relationships)? If nothing, please explain how it has not improved your social opportunities.
